# Adapting data science competencies by role and purpose: Voice AI

**DOI:** 10.3389/fdgth.2025.1610253

**Published:** 2025-10-21

**Authors:** David A. Dorr, Andrea Krussel, Rachel Hauck, Christie Jackson, Abhijeet Dalal, Steven Bedrick, Philip R. O. Payne, Bridge2AI-Voice Consortium, Yael Bensoussan, William Hersh

**Affiliations:** ^1^ Division of Informatics, Clinical Epidemiology, and Translational Data Science, Department of Medicine, Oregon Health & Science University, Portland, OR, United States; ^2^ Institute for Informatics, Data Science and Biostatistics, Washington University School of Medicine in St. Louis, St. Louis, MO, United States

**Keywords:** artificial intelligence, voice, speech, language, competency frameworks, machine learning, data science, personas

## Abstract

Competencies help define the skills and knowledge needed by learners. Often broad, educators integrate competencies to provide a framework for curricula or professional standards. For data science, the rate of change in the field, role variations, and specificity in key applications can be challenging. Our objective was to adapt general data science competencies for different learner roles in an emerging area: the clinical utility of Voice, Language, and Speech-based Artificial Intelligence/Machine Learning (AI/ML). Using a persona-inductive approach, we adapted competencies to support learners from varying professional and educational backgrounds and implemented these adaptations in a multi-institutional summer school. Results from these pilot efforts demonstrated feasibility, highlighted the importance of cross-role collaboration, and provided lessons for scaling to broader audiences. Our frameworks show that competency adaptation is necessary and practical in rapidly evolving AI domains.

## 1 Introduction

The rapid pace of data science and artificial intelligence (AI) development has outstripped the ability of education and training systems to keep up, especially in areas requiring the integration of multimodal data such as text, audio, images, and video (1, 2). Among these, voice and speech hold particular promise: advances in machine learning are enabling their use as digital biomarkers for detecting and monitoring a wide range of health conditions, from neurological and psychiatric disorders to cancers and cardiometabolic diseases (3, 4). However, realizing this potential requires not only technical expertise but also ethical oversight, clinical integration, and engagement from a broad set of stakeholders—including clinicians, researchers, administrators, policymakers, and patients (5).

**Table 1:** Personas.

Persona	Audience	Level	Product	Description
Clinical Investigators	Clinician–Speech language pathologists	Professional Trainee	Professional development module on data (e.g., CE req for licensure) Curriculum units focused on data, voice-based AI, and voice as a biomarker of health	Materials on naturalistic data collection practices, structure, and applications of voice-based data repositories, etc., with a hands-on exploratory activity involving a project dataset Materials on the basics of AI for voice disorders addressed, basic kinds of models (classification, regression, labeling, etc.), AI workflow (train/test cycle, interpretation of basic metrics), and ethical considerations, with a hands-on activity involving a project dataset as well as AI tools using that data
	Clinician—medical	Trainee—UME/GME Professional	Curriculum unit on voice as a biomarker, ML/AI (emphasis voice/audio/language analysis), voice/language data collection & management Products above, but framed as CME unit	Discuss what kinds of conditions have voice, basics of speech science, basics of acoustics, including content about bias issues & data use. Intro to basic concepts in ML/AI + audio processing, emphasis on issues of model evaluation (how to critically assess an article describing an AI system, etc.) and clinical implementation, inc. content on bias issues, etc. Introduce issues of dataset collection/curation/documentation (data sheets, etc.), stimulus design, evaluation, IP/privacy/consent issues, and also equity/bias issues. Using tablets/devices in clinical care/data collection—practical considerations, different populations (Peds vs. adults, diff. clinical conditions e.g., MCI, ASD, etc.)
Technical Experts	CS/ML/AI students	Undergraduate, graduate	Data-focused curriculum unit/materials. AI-focused curriculum unit/materials	Teach students about methods and best practices for creating and maintaining large-scale datasets, using the project dataset as an example (presumably looking at others)—discussions of design, curation, annotation/labeling, evaluation, governance, ethics, etc. Focus on core computational topics for voice-based AI –signal processing, acoustics, machine learning on audio data, etc. Use a specific clinically motivated example, such as hands-on use of the project dataset. Include content on ethical issues
	Clinical/translational research data managers & study coordinators	Professional	Workshop/seminar on voice data management	Working with multimodal/voice data—how to adapt existing data management pipelines to include sensor/voice data and integrate with existing data management infrastructure. Governance & storage issues
	CS/ML/AI researchers	Professional	Workshop/seminar series on speech language pathology, voice disorders, etc	Meant to introduce the clinical domain to a non-clinical technical audience, including technical details (specific computational/ acoustic details relating to specific disorders) and orientation to the dataset produced by the VBAI project

**Table 2:** Core elements of competencies’ adaptations for AI.

Domain	Competency (adapted for voice)
Basic knowledge of Data Science with a focus on AI	Data science life cycle, key model building techniques, computational methods for audio signal processing, ML model validation, and the potential of voice AI to address problems
Ethical considerations	Understand the FAIR and CARE frameworks and ethical concerns for AI in general and unique to voice
Data exploration and inference generation	Explore the adequacy of data for the unique feature extraction for voice
Evidence-based evaluation of AI tools	Evaluate the quality, accuracy, safety, contextual appropriateness, and biases for AI tools using voice
Implementations of AI tools	Understand the people, organization, and implementation issues related to AI tools for voice
Societal issues in AI	Focus on the broader landscape to build a virtuous learning cycle and address key issues in AI, especially using voice

These demands highlight a central challenge: traditional competency frameworks in biomedical informatics and AI education are often too rigid or slow to adapt to the speed of technological innovation. Emerging risks—including bias, reproducibility concerns, explainability, and safety—further underscore the need for new educational approaches that are flexible, inclusive, and ethically grounded (3).

This paper responds to these challenges by:

• Reviewing the current landscape of data science and AI competency frameworks, with particular attention to their application in healthcare and biomedical informatics.
• Identifying gaps in existing models when applied to rapidly evolving domains such as Voice AI, where technical advances and clinical applications are moving quickly, but educational frameworks lag.
• Our work within the Bridge2AI initiative, specifically within the Training, Recruitment, and Mentoring (TRM) group, provides a broader framework for cross-disciplinary, ethically grounded AI education.
• Presenting implications for future curriculum design, highlighting how adaptive, competency-based approaches can prepare learners to responsibly develop, implement, and use Voice AI tools in clinical and biomedical contexts and have the potential to provide a framework for other clinical AI domains.

## 2 Literature review

Meeting diverse learners' educational and professional needs is challenging, reflecting the field of AI's interdisciplinary and rapidly evolving nature. Competency development in Biomedical Informatics (BMI) is an excellent touch point; BMI is the “interdisciplinary field that studies and pursues the effective uses of biomedical data, information, and knowledge for scientific inquiry, problem-solving, and decision making, motivated by efforts to improve human health” (6). One of the primary objectives of informatics and data science is to develop technical proficiency among data science students, including foundational skills in machine learning (1). Clinicians must also know how to use and understand tools, including those with AI fields (7). Likewise, researchers must also be facile with data sources and tools for analyzing data in their modern work (8, 9).

To help prepare this diversity of learners for these needs, several groups have defined competencies in data science (10, 11) and AI for clinicians (12, 13). Aligning these competencies with learners' needs in application areas, like voice, is challenging due to the rapid development of new analysis methods and the strong desire to implement them in care. Goodman et al. (10) and Topol (14) emphasize readiness challenges for clinicians adopting AI tools. Our work builds upon these frameworks by focusing on personas, iterative adaptation, and curricular implementation. AI competency, in contrast, denotes practical proficiency in using, engaging, interacting, developing, or managing AI systems and specific tasks that are relevant in real-world contexts (15).

Historical approaches to align competency models have included consensus-based deductive methods from experts and educators in the field. The deductive approach requires substantial time, and new methods and requirements often outpace the updates to the frameworks. However, more pragmatic approaches to data science competencies have started to take an inductive approach focused on the broader competency elements targeted to specific areas. For instance, training in new models of AI/ML may include broad concepts like “Data exploration and inference generation,” with key principles supplemented with self-directed and interactive problem-solving. The benefit of the inductive approach is that it matches knowledge of adult learning standards and enables lifelong learning.

Rapid development in Machine Learning has been a constant, but new and highly complex models have opened a brand-new set of requirements for learners. This rapid development includes incorporating multimodal data, new coding approaches, and new application areas. Developing, testing, and implementing ML/AL models for the voice, speech, and language continuum is a near-perfect example of this triumvirate of new capabilities. Extraction of key information from voice and respiration has shown promise in multiple health conditions across otolaryngology, neurology, psychiatry, infectious disease, and cardiology. The techniques to process these models have evolved quickly, and the ability to change the voice into language can be near instantaneous, allowing for rapid, complex model development. Large Language Models—already transforming language-based analyses—show promise in concept and feature extraction across many data types, including voice and speech. One key aspect of these models is the strong demand for immediate implementation in clinical care, even when evidence for their safe and effective use is limited (3).

These new capabilities come with new risks and biases. While the issues of fairness, accuracy, verification, explainability, and safety have long been known, the behavior of the models has changed the manifestations and implications of these risks. For instance, the ability to emulate any voice or image makes verifiability challenging; the persistence of large models across federated spaces with active learning significantly impacts data retention; and the promiscuous incorporation of biased data in unpredictable models presents major ethical concerns.

As part of an effort to build an ethically sourced dataset for training models across the Voice AI continuum, the Voice as a Biomarker of Health (NIH) program, one of four data generation projects (DGPs) funded by the National Institute of Health's Bridge2AI program, has been launched. Voice, speech, and breathing sounds can reveal valuable information about a patient's health. With advances in AI, these audio signals are being studied as potential digital biomarkers to support early detection of conditions ranging from voice disorders and neurological diseases to head and neck cancers and diabetes. Clinicians across multiple fields—including otolaryngology, neurology, speech-language pathology, and internal medicine—bring complementary expertise critical to advancing this emerging area of research and practice (4).

## 3 Methods

The Bridge2AI initiative has emphasized the importance of curriculum innovation to close gaps in AI education. Its TRM working group developed a cross-disciplinary curriculum to address deficits in accessibility, reproducibility, integration with clinical practice, and stakeholder engagement. Their model prioritizes ethically sourced data, fosters collaboration across domains, and cultivates professional skills that support accountability and adaptability in biomedical and healthcare settings (16). The model provides a valid comparison point for our work on the Voice AI TRM team, which has been tasked with building competency-based education programs to teach multiple personas how to develop and implement standardized methods for voice data collection to fuel scientific discovery and ethical development of AI/ML models.

1. We used a persona-based inductive approach to adapt existing ***core data science competencies*** to the domain of Voice AI. First, we reviewed and synthesized multiple frameworks, including the EDISON Data Science Framework (17), AMIA informatics competencies (9), and recent AI competencies for clinicians (12, 13).
2. We then developed ***personas*** based on the NIH CD2H framework, focusing on two broad categories: (1) clinical learners (e.g., clinicians, speech-language pathologists, medical trainees) and (2) technical learners (e.g., informaticians, data scientists, engineers) illustrated in Table 1 below. Personas were refined through expert consensus with members of the NIH Bridge2AI-Voice consortium.
3. **Table 2 reveals how the *Competencies were adapted*** iteratively in working groups, with feedback from interdisciplinary educators and domain experts. Adapted competencies included foundational knowledge (e.g., data lifecycle, AI model building) and contextual considerations (e.g., ethics, FAIR/CARE frameworks, societal implications).
4. We implemented the adapted competencies in a multi-site summer school program in 2024 across four institutions (OHSU, Washington University in St. Louis, Weill Cornell Medicine, and the University of South Florida) to test them. The program included didactic instruction, workshops, and a culminating hackathon event where ***interdisciplinary teams addressed challenges*** and integrated their AI competencies with clinical voice datasets. The evaluation included questions surrounding program experience, learner feedback, and feasibility across sites.

We have illustrated the detailed methods below:

a. Core Data Science Competencies
  To create the adaptation, we reviewed the data science competencies defined by several initiatives, including the Data Science Initiative, an NIH program that seeks to build health science capacity in Africa. Several authors of this work are investigators on this project and have been building competency-based data science programs in partnership with universities across Sub-Saharan Africa. The training program integrates three core interdisciplinary areas: Computer Science/Informatics, Statistics/Mathematics, and Domain-specific knowledge with diverse mentorship from experts across basic sciences to community-based research initiatives (18). We utilized additional adaptations from EDISON, which identified several key competencies, including Data Analytics, which encompasses statistical methods, machine learning, and business analytics, all of which are essential for extracting insights and making data-driven decisions; engineering competencies, including software development and infrastructure management; and competencies in scientific or research methods to ensure data-driven research meets high standards of validity and reliability (17). IBM Analytics was also utilized and identified the following competencies for their data science apprenticeship model: Statistics and programming foundation, data science foundation, data preparation, model building, model deployment, big data foundation, and leadership and professional development.
  The list below highlights the six high-level competencies identified from over 60 individual competencies across twelve domains. Cognizant of the strong pressure for use of these models, we leveraged a paper by Russell et al. (12) that focused on the end-users of AI development: clinicians. After conducting semi-structured interviews with 15 healthcare experts, six clinical competencies and 25 sub-competencies were identified. This focus—while practical—also enables easy adaptation. For instance, the “basic knowledge” of techniques may be deep and hands-on for data scientists, while clinicians may learn what to look for in descriptions of model development. Similarly, core foundational ethical considerations can be with specific adaptations for implementations and developers related to their professional roles.
  • Basic knowledge of data science techniques, including AI
  • Ethical considerations
  • Data exploration and inference generation
  • Evidence-based evaluation
  • Implementation of tools
  • Societal issues
b. Personas: Defining Key Learner Types and Roles
  We used personas to adapt the competencies further and apply them to specific areas. Personas are representations of potential groups of learners with information about their general needs and goals. We based our personas on roles created for clinical and translational science through the National Center for Data to Health (CD2H). We focused on two categories and four roles: clinical Investigators, including experts and trainees, and technical roles, including informatics and data science experts and trainees. We then divided these initial four roles into Voice AI-specific groupings and identified vital needs and curricular products for these groups. For instance, clinician professionals such as speech-language pathologists may be the front line for collecting data for AI use, understanding the results, and describing the impacts of AI models. In contrast, clinician trainees need a broader sense of Voice AI applications and AI workflow. Technical learners need a more fundamental approach to ML/AI development and testing specific to Voice AI. They also require a background in the clinical problems and their current diagnosis, prognosis, and treatment to develop targeted, practical solutions. All groups require deep core ethical discussions and implications, carefully considering role-based needs.
c. Adapted Competencies
  Once we had developed the personas, we engaged with experts and educators from the Voice AI collaborative to adapt each core competency to the needs of the learners. For each, we took the core elements of the competencies and iterated on the common needs of learners and the specific elements required for voice. For instance, basic knowledge includes the data science lifecycle and core model building, as well as how to learn about particular methods and features for the voice. In addition, guidance is given on how to review the potential applications and current evidence for the use of AI specific to voice. Ethical concerns have a foundation in key frameworks, such as the need to make data and models Findable, Accessible, Interoperable, and Reusable (FAIR), as well as the core concerns related to voice, especially for communities facing historical discrimination, such as Indigenous peoples. Voice AI can potentially extend the historical theft of voice and language; these considerations require frameworks like CARE—working with affected communities for Community benefit, granting Authority to control, defining Responsibility, and exploring the Ethical implications. The adaptations are intended to give deeper expertise in this area and empower learners through the inductive model by giving them the skills and framework to step through for other areas over time.
d. Curricular Design: Adaptation for topic: Voice AI
  Table 3 demonstrates the curricula developed from the adapted competencies, including specific, available curricular resources (links, in black) in informatics and data science.
e. Cross-pollination of roles: team challenges

**Table 3:** Curricular resources.

Domain	Competency (adapted for voice)	Available Curricular Resources
Basic knowledge of AI	Data science life cycle, key model building techniques, computational methods for audio signal processing, ML model validation, and the potential of voice AI to address problems	AI in Medicine & Medical Education: Critical Issues and Potential Solutions - William Hersh, MD: This lecture defines the major types of AI and their applications, successes, and limitations in biomedicine. Digital Health Leadership and Clinical AI—Philip Payne, PhD, and Andrea Krussel, MA, PhD Candidate: This lecture discusses AI in the context of its application to health and healthcare, such as building and operating an AI-enabled Learning Health System (LHS)
Ethical considerations	Understand FAIR (Findable, Accessible, Interoperable, Reusable) and CARE (Collective benefit, Authority to control, Responsibility, and Ethics) framework and ethical concerns for AI in general and unique to voice	How to be FAIR and CARE in AI—David Dorr, MD, MS: This lecture provides in-depth definitions of each component of the FAIR and CARE principles; emphasizes their importance in the context of Voice AI and AI in general; describes the history and current state of FAIR and CARE collaboratives; and explains how to teach and implement FAIR and CARE
Data exploration and inference generation	Explore the adequacy of data for the unique feature extraction for voice	*Team-based challenges, below* Intro to Bridge2AI Voice Data—Alexandros Sigaras, MS, and Alistair Johnson, DPhil This lecture explores the data collection lifecycle of the Bridge2AI Voice dataset; explains standards for sharing voice data, such as FHIR and BIDS; highlights the Bridge2AI open-source repository and data dictionary; and discusses future directions for data dissemination
Evidence-based evaluation of AI tools	Evaluate the quality, accuracy, safety, contextual appropriateness, and biases of AI tools using voice	Voice-Based Biomarkers Through the Lens of Validity—Steven Bedrick, PhD: This lecture defines biomarkers and validity in the context of biomarkers; describes how voice biomarkers are validated; discusses considerations for machine learning and validity; and explores case studies of voice biomarkers
Implementations of AI tools	Understand the people, organization, and implementation issues related to AI tools for voice	Voice AI for Low-Resource Healthcare Settings—James Anibal, PhD Candidate: This lecture presents an overview of two studies focused on enhancing the accessibility of voice AI in low resource healthcare settings: 1) use of multimodal audio data to identify YouTube videos with COVID-19-positive speakers (data collection, data analysis, results, challenges) and 2) feasibility of clinical AI with self-reported health information and voice data (study workflow, key questions, and future directions for research)
Societal issues in AI	Focus on the broader landscape to build a virtuous learning cycle and address key issues in AI, especially using voice	The AI Life Cycle from a DEI Perspective—Maria Powell, PhD: This lecture describes key components of the lifecycle of an AI project and how to apply a diversity, equity and inclusion (DEI) mindset to each stage of the process; explains how to identify practical strategies for promoting DEI and accessibility in the design, development and deployment of AI projects, including team assembly, problem formulation, protocol development, and community engagement

One key aspect of this approach is that learning must be cross-pollinated across roles; in essence, learning to work as a team so that the specific learned competencies of each group can complement each other. To this end, we developed team challenges to allow interdisciplinary teams to work together to improve their understanding of others' competencies. Table 4, below, highlights examples of challenges and critical competencies they address. Teams must communicate and problem-solve effectively; success is defined, in part, by recognizing the diverse experiences that each brings. Thus, the personas have key areas to explain, offering their expertise to ensure the team achieves the best possible outcomes. In exploring data science techniques for key clinical areas, clinicians can help define the need and guide the interpretation of results. At the same time, the technical personas can decide on the ensemble method, identify features related to the need, and explain the technical aspects of the results. Similarly, for ethical concerns about identifying current and former smokers through voice analysis, clinicians can help give examples of historical biases and risks to health. At the same time, the technical team can consider potential requirements for the accuracy and reliability of these models and their possible implementations.

**Table 4:** Challenges and personas.

Competency	Challenge	Persona roles
Data science/AI techniques	Develop an ensemble method for classifying a Voice-Speech-Language-related clinical diagnosis	Clinical: Choose diagnosis, interpret results. Technical: choose ensemble method, evaluate, and describe the results
Ethical concerns	Smoking Status: Can an AI model be developed to predict past smoking status? What are the ethical implications of predicting a social behavior that the patient could report themselves as a response to a simple question?	Clinical: reflect on the impact of automated smoking status detection. Technical: identify the reliability and accuracy of potential features and their impact
Data exploration and inference generation	True Controls: Exploration of the “normal voice” concept—does this exist?	Clinical: Define “normal' and potential. Technical: Explore characteristics of features that define health conditions and their alternatives

## 4 Results

To address the growing need to apply AI techniques to acoustic data (voice and sounds), we established a new training activity: an interdisciplinary (medical, nursing, engineering, and science) summer school program for undergraduate and graduate students from four different universities already funded for a Data Generation Project (DGP) “Voice as a Biomarker of Health” as part of the NIH Bridge2AI consortium. The training activity aimed to train students from diverse backgrounds to create computer programs and machine-learning models that use acoustic data for medical applications. The training activities involved identifying areas of unmet medical needs where voice or sounds may help create new solutions, acquiring, managing, and analyzing existing acoustic datasets, and building, testing, and validating AI models that leverage state-of-the-art AI methods (e.g., deep learning), creating user interfaces and if feasible, deploying the applications in a real-world setting.

Our application for supplemental funding was accepted, and as of this writing, the inaugural Voice AI summer school program launched in 2024 at Oregon Health and Science University (OHSU), Washington University in St. Louis, Weill Cornell Medicine, and the University of South Florida (USF). 50 graduate and undergraduate students (selected from a highly competitive and deep pool of interested applicants) participated across the four sites in a 5-week-long course culminating in a hackathon event. The curriculum included an online platform for individual learning, didactic in-person lectures, and workshops with case studies using voice datasets. In the hackathon event, interdisciplinary teams (clinical and informatics students) competed against each other to develop the best models to answer tangible clinical questions using voice data from the Voice DGP. Developed AI models have been made publicly available.

## 5 Discussion

Competency frameworks help develop curricula and define professional needs. Still, in data science and AI, the rate of change and shifts in paradigms make deductive approaches to competencies challenging. This manuscript describes a method for taking broad competency areas and refining them for specific new areas of analysis (Voice-Speech-Language). We then discuss how we developed specific curricular adaptations to help learners understand the specific concerns of Voice AI while still understanding the core framework needed to ethically develop, evaluate, and implement models for any particular challenge.

Limitations of this work include its focus on one domain (Voice AI) and short-term evaluation in a summer program. Longitudinal assessment of learner outcomes and expansion to additional modalities are the necessary next steps.

## 6 Conclusion

Adapting data science competencies to emerging domains such as Voice AI requires theoretical grounding and pragmatic curricular design. By combining inductive adaptation with role-based personas, we developed a competency framework that flexibly addresses the needs of learners from different backgrounds. Our pilot summer school demonstrated feasibility and revealed pathways for scaling to broader audiences, including clinicians, data scientists, and interdisciplinary teams. While limited to one application domain, this work provides a transferable model for adapting competencies in other rapidly evolving AI subfields. Future directions include multi-institutional testing, expansion to additional modalities, and long-term evaluation of educational outcomes.

## Data Availability

Publicly available datasets were analyzed in this study. This data can be found here: PhysioNet Repository, Bridge2AI-Voice Dataset v1.1, doi: 10.13026/6rcx-na48, https://physionet.org/content/b2ai-voice/2.0.1/.

## Group member of Bridge2AI-Voice Consortium

University of South Florida, Tampa, FL, US: Yael Bensoussan. Weill Cornell Medicine, New York, NY, USA: Olivier Elemento. Weill Cornell Medicine, New York, NY, USA: Anais Rameau. Weill Cornell Medicine, New York, NY, USA: Alexandros Sigaras. Massachusetts Institute of Technology, Boston, MA, USA: Satrajit Ghosh. Vanderbilt University Medical Center, Nashville, TN, USA: Maria Powell. University of Montreal, Montreal, Quebec, Canada: Vardit Ravitsky. Simon Fraser University, Burnaby, BC, Canada: Jean Christophe Belisle-Pipon. Oregon Health & Science University, Portland, OR, USA: David Dorr. Washington University in St. Louis, St. Louis, MO, USA: Phillip Payne. University of Toronto, Toronto, Ontario, Canada: Alistair Johnson. University of South Florida, Tampa, FL, USA: Ruth Bahr. University of Florida, Gainesville, FL, USA: Donald Bolser. Dalhousie University, Toronto, ON, Canada: Frank Rudzicz. Mount Sinai Hospital, Sinai Health, University of Toronto, Toronto, ON, Canada: Jordan Lerner-Ellis. Boston Children's Hospital, Boston, MA, USA: Kathy Jenkins. University of Central Florida, Orlando, FL, USA: Shaheen Awan. University of South Florida, Tampa, FL, USA: Micah Boyer. Oregon Health & Science University, Portland, OR, USA: William Hersh. Washington University in St. Louis, St. Louis, MO, USA: Andrea Krussel. Oregon Health & Science University, Portland, OR, USA: Steven Bedrick. UT Health, Houston, TX, USA: Toufeeq Ahmed Syed. University of South Florida, Tampa, FL, USA: Jamie Toghranegar. University of South Florida, Tampa, FL, USA: James Anibal. New York, NY, USA: Duncan Sutherland. University of South Florida, Tampa, FL, USA: Enrique Diaz-Ocampo. University of South Florida, Tampa, FL, USA: Elizabeth Silberhoz Boston Children's Hospital, Boston, MA, USA: John Costello. Vanderbilt University Medical Center, Nashville, TN, USA: Alexander Gelbard. Vanderbilt University Medical Center, Nashville, TN, USA: Kimberly Vinson. University of South Florida, Tampa, FL, USA: Tempestt Neal. Mount Sinai Health, Toronto, ON, Canada: Lochana Jayachandran. The Hospital for Sick Children, Toronto, ON, Canada: Evan Ng. Mount Sinai Health, Toronto, ON, Canada: Selina Casalino. University of South Florida, Tampa, FL, USA: Yassmeen Abdel-Aty. University of South Florida, Tampa, FL, USA: Karim Hanna. University of South Florida, Tampa, FL, USA: Theresa Zesiewicz. Florida Atlantic University, Boca Raton, FL, USA: Elijah Moothedan. University of South Florida, Tampa, FL, USA: Emily Evangelista. Vanderbilt University Medical Center, Nashville, TN, USA: Samantha Salvi Cruz. Weill Cornell Medicine, New York, NY, USA: Robin Zhao. University of South Florida, Tampa, FL, USA: Mohamed Ebraheem. University of South Florida, Tampa, FL, USA: Karlee Newberry. University of South Florida, Tampa, FL, USA: Iris De Santiago. University of South Florida, Tampa, FL, USA: Ellie Eiseman. University of South Florida, Tampa, FL, USA: JM Rahman. Boston Children's Hospital, Boston, MA, USA: Stacy Jo. Hospital for Sick Children, Toronto, ON, Canada: Anna Goldenberg.
